# Supplementary material for: Integrated Multiomics Analysis of Salivary Exosomes to Identify Biomarkers Associated with Changes in Mood States and Fatigue
Source: Int J Mol Sci. 2022 May 9;23(9):5257. doi: 10.3390/ijms23095257 (PMC9105576; doi:10.3390/ijms23095257)
Supplement: Supplementary file 1 [file ijms-23-05257-s001.zip › ijms-1662519-supplementary.pdf]

Supplementary Information for:

Integrated multi-omics analysis of salivary exosomes to identify biomarkers associated with changes in mood states and fatigue

Whitaker Cohn[1], Chunni Zhu[1], Jesus Campagna[1], Tina Bilousova[1], Patricia Spilman[1], Bruce Teter[1], Feng Li[2], Rong Guo[3], David Elashoff[3], Greg Cole[4], Alon Avidan[4], Kym Francis Faull[5], Julian Whitelegge[5], David T.W. Wong[2], Varghese John[1*]

Supplementary Figures

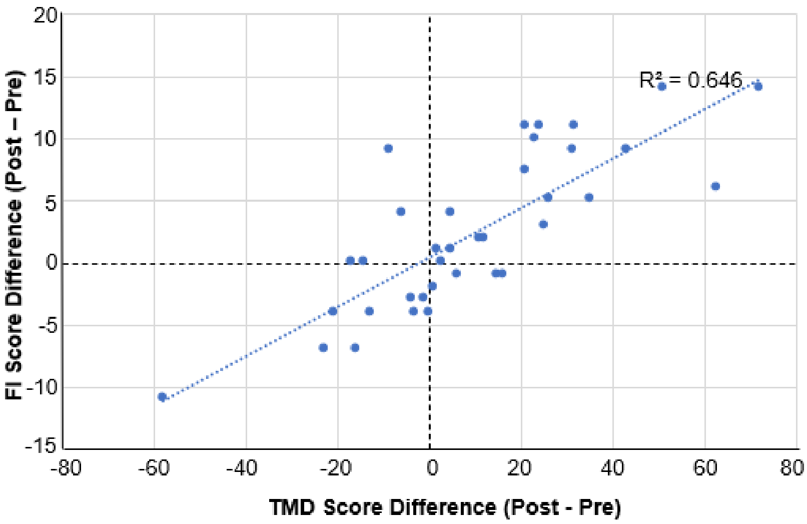

Supplementary Figure S1. *Difference in Total Mood Disturbance (TMD) positively correlates with difference in Fatigue-Inertia (FI) subscale as assessed by the Profile of Mood States (PoMS).* The difference in subject FI score in PoMS (y-axis) is shows a moderate correlation (R^2=0.646, p = 0.00002) with difference in subject TMD score (x-axis).

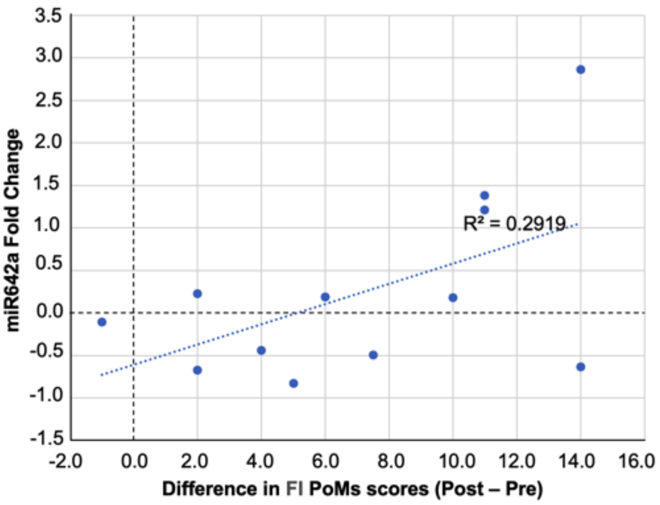

Supplementary Figure S2. *miR642a identified in the Discovery group correlates with Fatigue-Inertia (FI).* miR642a fold change (y-axis) shows a weak positive correlation (R^2 = 0.29, R(12) = 0.54, p = 0.0699) with FI difference in Discovery group participants after a work shift. The data only include subjects in which both pre- and post-work qPCR results were obtained.

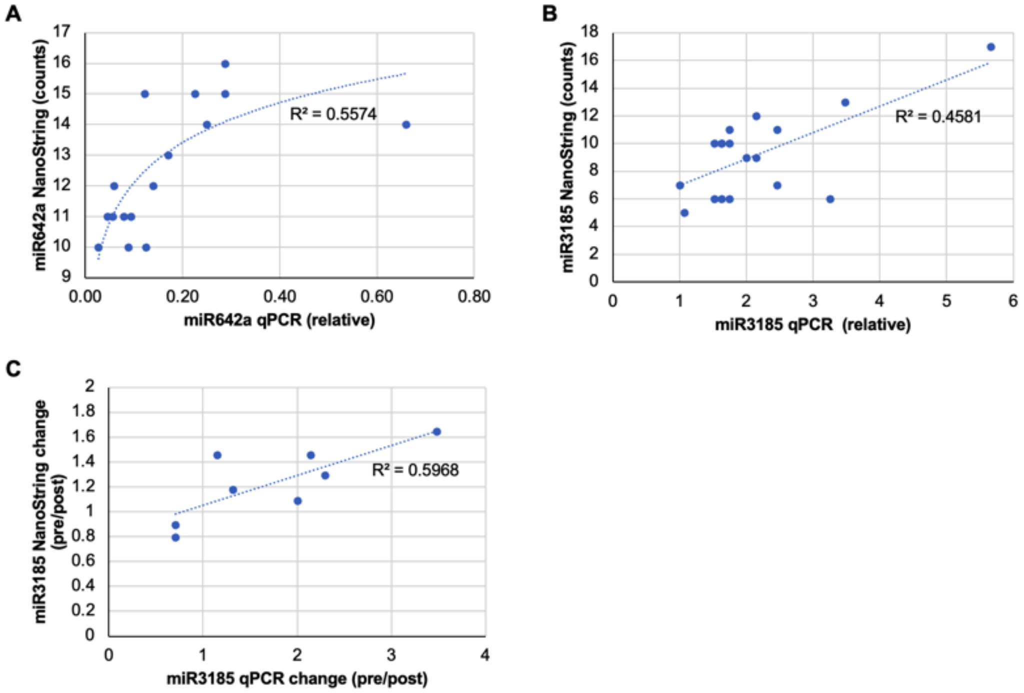

Supplementary Figure S3. *Verification of Discovery group NanoString miRNA measurements using qPCR.* Correlations for abundances of miRNAs in the Discovery group as determined by NanoString (y-axis) and qPCR (x-axis) in the same RNA samples, as well as R^2, r, and p values are shown for: (A) miR642a, where R^2 = 0.56, R(16) = 0.61, and p = 0.012 (Nanostring levels with less than 9 counts were not reliably detectable by qPCR and were excluded); (B) miR3185 in Discovery Group samples where R^2 = 0.46, R(18) = 0.68, and p = 0.0019 (data shown for samples from which miR3185 was reliably detected are included); (C) miR3185 change between pre- and post-work shift where R^2 = 0.60, r (8) = 0.77, and p = 0.0254 (data shown for samples from which miR3185 was reliably detected both pre- and post-work are included).

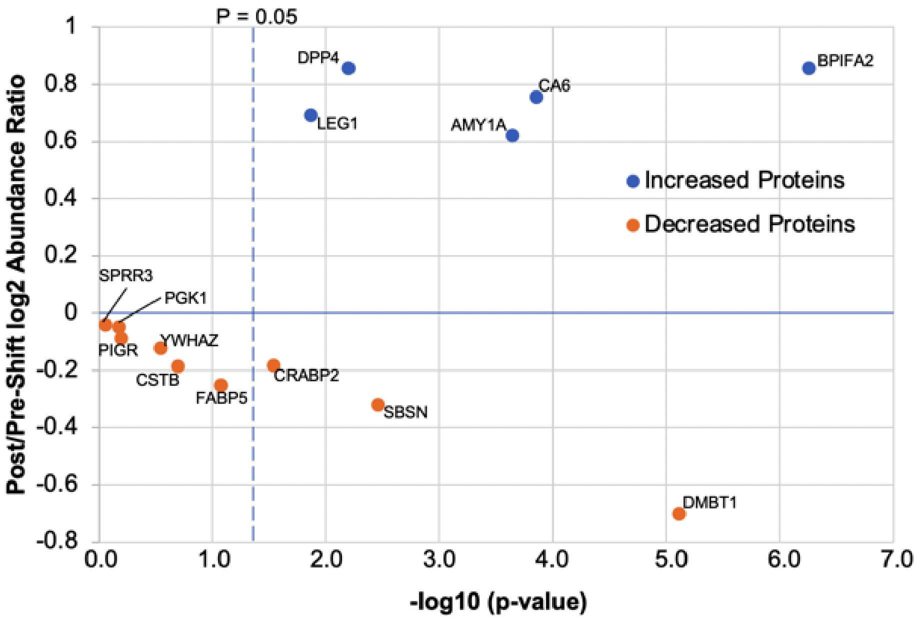

Supplementary Figure S4. *Integration of Test, Discovery and Validation group data reveals proteins altered post-work shift.* Differences in the abundances of 14 proteins (AMY1A, BPIFA2, CA6, CSTB, DMBT1, DPP4, FABP5, LEG1, PIGR, PGK1, SPRR3, YWHAZ, CRABP2, SBSN) present in the salivary exosomes of Test, Discovery, and Validation group participants pre- and post-work shift are displayed via volcano plot. The log2 (abundance ratio: post-work/pre-work) is plotted against -log10(p-value).

Supplementary Tables

Supplementary Table S1. *Analysis of PoMS scores pre- and post- work shift.*

Subscale	Work	Mean	Std Dev	Median	Lower Quartile	Upper Quartile	Minimum	Maximum	Wilcoxon Signed Rank Test (p value)
AH	Pre	16.13	4.73	14.5	13	17.5	12	32	0.1748
	Post	17.44	5.45	16	13	20.5	12	33	
CB	Pre	10.49	2.83	10	9	12	7	18	0.0033
	Post	11.88	3.37	11	10	12.5	8	22	
DD	Pre	17.49	4.79	15.5	14	19.5	13	32	0.7476
	Post	18.19	5.46	17	14	21.5	13	31	
FI	Pre	15.68	4.59	14.5	12.5	18.5	9	26	0.0393
	Post	18.08	5.21	17	13.5	22	9	28	
TA	Pre	16.88	5.43	16	12	19	10	33	0.1783
	Post	17.97	5.76	18	14	20	9	33	
TMD	Pre	55.51	22.2	48.25	38.5	67	22	105	0.02
	Post	65.46	24.31	63.5	46	78	31	121	
VA	Pre	21.14	5.4	22	17.5	25	10	33	0.0047
	Post	18.11	5.87	16.5	14	21.5	8	32	

AH - Anger, Hostility; CB - Confusion, Bewilderment; DD - Depression, Dejection; FI - Fatigue, Inertia; TA - Tension, Anxiety; TMD - Total Mood Disturbance; VA - Vigor, Activity

Supplementary Table S2. *Test Group pan-exosome marker immunoprecipitated proteins of interest and corresponding miRNA that may regulate the gene encoding the protein.*

Gene Symbol	Description	Protein Fold Change: Post- / Pre-Work	Protein Fold ChangeP-Value	Associated miRNA from NanoString Analysis	miRNA Fold Change: Post- / Pre-Work	miRNA Fold Change P-Value
HSPD1	60 kDa heat shock protein	9.36	0.06			
GIMAP8	GTPase IMAP family member 8	4.34	0.20			
RPS27A	Ubiquitin-40S ribosomal protein S27a [n]	3.07	0.16			
DSC1	Desmocollin-1 [m]	2.62	0.10			
BPIFA2	BPI fold-containing family A member 2 [n]	1.91	0.02			
AMY1A	Alpha-amylase 1 [n,c]	1.82	0.40			
MUC7	Mucin-7 [n]	1.77	0.26			
DSG3	Desmoglein-3 [m]	1.57	0.30	hsa-mir-1304-3p	1.88	0.02
PGK1	Phosphoglycerate kinase 1 [n]	-1.51	0.11			

CSTB	Cystatin-B [n,c]	-1.53	0.18			
IGLL5	Immunoglobulin lambda-like polypeptide 5	-1.88	0.27			
PPARD	Peroxisome proliferator-activated receptor delta	-1.90	0.48			
EEF1A2	Elongation factor 1-alpha 2 [n]	-1.96	0.06			
PIGR	Polymeric immunoglobulin receptor [m,n,c]	-2.15	0.49	hsa-mir-532-3p<br>hsa-mir-642a-5p	1.27<br>1.24	0.02<br>0.01
HIST1H2BN	Histone H2B	-2.24	0.27			
DMBT1	Deleted in malignant brain tumors 1 protein [n,c]	-2.24	0.23			
LYZ	Lysozyme C [n,c]	-2.35	0.19	hsa-mir-873-3p	1.24	0.03
RAC1	Ras-related C3 botulinum toxin substrate 1 [m,c]	-2.35	0.36	hsa-mir-381-3p<br>hsa-mir-374b-5p	1.30<br>1.29	0.01<br>0.05
PKM	Pyruvate kinase [n]	-2.40	0.13	hsa-mir-221-3p<br>hsa-mir-765	0.66<br>0.78	0.01<br>0.05
SPRR3	Small proline-rich protein 3	-2.58	0.05			

[m] = Membrane Protein; [n] = Experimentally identified using neuron-specific marker (CD171); [c] = Relevant in Chronic Fatigue Syndrome

Supplementary Table S3. *Gene Set Enrichment Gene Ontology (GO) cellular component analysis of Test group proteins*

**Cellular Component (Gene Ontology)**				
**GO-term**	**Description**	**Count in Network**	**Strength**	**False Discovery Rate**
GO:0005615	Extracellular space	92 of 3195	0.73	5.76E-55
GO:0070062	Extracellular exosome	80 of 2099	0.86	1.89E-52
GO:0031982	Vesicle	89 0f 3879	0.64	4.46E-44
GO:0030141	Secretory Granule	42 of 845	0.97	6.59E-27
GO:0034774	Secretory Granule Lumen	25 of 324	1.16	4.05E-19

Supplementary Table S4. *Proteins found in neuron-derived CD171 immuno-precipitated exosomes from a single Test group participant.* Proteins in blue were also found in pan-exosomal marker immunoprecipitated exosomes.

Accession	Gene Symbol	Description	Exp. q-value: Combined	Sum PEP Score
Q9HC84	MUC5B	Mucin-5B	0	151.144
Q8TDL5	BPIFB1	BPI fold-containing family B member 1	0	96.533
P04745	AMY1A	Alpha-amylase 1	0	51.69
P60709	ACTB	Actin, cytoplasmic 1	0	40.786
P01833	PIGR	Polymeric immunoglobulin receptor	0	39.443
Q96DR5	BPIFA2	BPI fold-containing family A member 2	0	38.252
P23280	CA6	Carbonic anhydrase 6	0	36.831
P13646	KRT13	Keratin, type I cytoskeletal 13	0	34.795
Q96DA0	ZG16B	Zymogen granule protein 16 homolog B	0	33.032
Q8N4F0	BPIFB2	BPI fold-containing family B member 2	0	28.386
P01037	CST1	Cystatin-SN	0	26.446
Q9UGM3-9	DMBT1	Deleted in malignant brain tumors 1 protein	0	24.278
A0A286YEY1	IGHA1	Immunoglobulin heavy constant alpha 1 (Fragment)	0	20.792
P12273	PIP	Prolactin-inducible protein	0	19.316
P19013	KRT4	Keratin, type II cytoskeletal 4	0	17.076
P04406	GAPDH	Glyceraldehyde-3-phosphate dehydrogenase	0	16.443
P01036	CST4	Cystatin-S	0	15.874
P02814	SMR3B	Submaxillary gland androgen-regulated protein 3B	0	15.701
P81605-2	DCD	Dermcidin	0	12.922
P01834	IGKC	Immunoglobulin kappa constant	0	11.781
Q7Z794	KRT77	Keratin, type II cytoskeletal 1b	0	10.86
Q6MZM9	PRR27	Proline-rich protein 27	0	9.224
P28325	CST5	Cystatin-D	0	8.864
P15924	DSP	Desmoplakin	0	8.379
P02647	APOA1	Apolipoprotein A-I	0	7.217
P01034	CST3	Cystatin-C	0	6.738
P14923	JUP	Junction plakoglobin	0	6.599
P31025	LCN1	Lipocalin-1	0	6.046
Q02413	DSG1	Desmoglein-1	0	6.023
P02768	ALB	Serum albumin	0	5.99
P62805	HIST1H4A	Histone H4	0	5.36
Q99935	OPRPN	Opiorphin prepropeptide	0	5.086
P61626	LYZ	Lysozyme C	0	4.832
Q9NP55	BPIFA1	BPI fold-containing family A member 1	0	4.647
P05109	S100A8	Protein S100-A8	0	4.245
P06702	S100A9	Protein S100-A9	0	4.234

P10599	TXN	Thioredoxin	0	4.04
P47929	LGALS7B	Galectin-7	0	3.967
P31151	S100A7	Protein S100-A7	0	3.946
P52209	PGD	6-phosphogluconate dehydrogenase, decarboxylating	0	3.905
P25311	AZGP1	Zinc-alpha-2-glycoprotein	0	3.745
Q2I0M4	LRRC26	Leucine-rich repeat-containing protein 26	0	3.709
F8W1S1	KRT74	Keratin, type II cytoskeletal 74	0	3.626
Q08554	DSC1	Desmocollin-1	0	3.461
P09211	GSTP1	Glutathione S-transferase P	0	3.285
P40394-2	ADH7	Alcohol dehydrogenase class 4 mu/sigma chain	0	3.225
Q06830	PRDX1	Peroxiredoxin-1	0	3.17
I3L3D5	PFN1	Profilin (Fragment)	0	3.038
Q9NZT1	CALML5	Calmodulin-like protein 5	0	2.802
P06312	IGKV4-1	Immunoglobulin kappa variable 4-1	0	2.795
P04080	CSTB	Cystatin-B	0	2.725
A0M8Q6	IGLC7	Immunoglobulin lambda constant 7	0	2.686
Q8TAX7	MUC7	Mucin-7	0	2.677
P62979	RPS27A	Ubiquitin-40S ribosomal protein S27a	0	2.444
P63104	YWHAZ	14-3-3 protein zeta/delta	0	2.401
P04792	HSPB1	Heat shock protein beta-1	0	2.193
P30041	PRDX6	Peroxiredoxin-6	0.004	1.976
K7ES00	H3F3B	Histone H3.3 (Fragment)	0.004	1.809
P59665	DEFA1	Neutrophil defensin 1	0.007	1.705
G8JLG2	CDSN	Corneodesmosin	0.007	1.698
A0A0U1RRH7	H2A	Histone H2A	0.007	1.62
P14618	PKM	Pyruvate kinase	0.007	1.51
P04083	ANXA1	Annexin A1	0.006	1.495
P00558	PGK1	Phosphoglycerate kinase 1	0.006	1.418
P35030	PRSS3	Trypsin-3	0.009	1.357
Q5T750	XP32	Skin-specific protein 32	0.009	1.297
Q5T749	KPRP	Keratinocyte proline-rich protein	0.009	1.269
Q495G5	MMAA	Methylmalonic aciduria type A protein, mitochondrial	0.009	1.254
Q05639	EEF1A2	Elongation factor 1-alpha 2	0.009	1.236
P01871-2	IGHM	Immunoglobulin heavy constant mu	0.009	1.18
A0A087WY73	PRR4	Proline-rich protein 4	0.014	1.168
P06870	KLK1	Kallikrein-1	0.014	1.106

*Proteins highlighted in blue were also identified from the immunoprecipitation using exosomal cell surface markers (CD9, CD63, CD81)

Supplementary Table S5. *Significantly changed Test group miRNAs.* The 22 miRNAs found to be changed in the Test group are listed at the top, while the 12 miRNAs in the Test group that were also later found to be changed in the Discovery and/or Validation groups are listed at the bottom.

miRNA	Fold Change	p-value
525-3p	1.94	0.003
545-3p	1.81	0.032
1252-5p	1.77	0.01
376a-2-5p	1.42	0.047
3690	1.34	0.014
4536-3p	1.28	0.017
551b-3p	1.24	0.044
7e-5p	1.23	0.024
196a-5p	-1.22	0.038
203a-5p	-1.23	0.001
7i-5p	-1.26	0.021
21-5p	-1.27	0.037
626	-1.28	0.021
7f-5p	-1.39	0.028
885-3p	-1.39	0.043
7b-5p	-1.4	0.017
487b-3p	-1.43	0.023
548j-5p	-1.45	0.044
221-3p	-1.53	0.012
555	-1.54	0.041
152-5p	-1.57	0.015
1204	-1.86	0.015
1304-3p	1.88	0.018
1296-5p	1.58	0.018
135b-5p	1.35	0.027
513c-5p	1.33	0.038
381-3p	1.3	0.014
374b-5p	1.29	0.048
532-3p	1.27	0.016
1257	1.27	0.028
642a-5p	1.24	0.014
873-3p	1.24	0.034
765	-1.29	0.047
381-5p	-1.48	0.037

Supplementary Table S6. *Raw qPCR measures for miR3185 and miR642a in individual subject's samples from which the miRNA was reliably detected.*

SUBJECT ID	miR3185 (relative)	SUBJECT ID	miR642a
3A	2.14	3A	0.08
3B	1.62	3B	0.09
4A	2.00	4A	0.03
4B	1.74	4B	0.05
8A	1.07	8B	0.06
8B	1.52	9A	0.09
11B	1.00	9B	0.23
11A	2.14	18A	0.25
17A	1.74	18B	0.14
18A	3.48	19B	0.13
18B	1.52	28B	0.29
25A	2.46	29A	0.29
28A	3.25	29B	0.06
28B	1.62	36A	0.17
29A	1.74	36B	0.66
29B	2.46	37B	0.12
36A	5.66		
36B	1.62		

Supplementary Table S7. *Proteins quantified in pan-exosomal marker immunoprecipitated exosomes from Discovery group participants*

Accession	Gene Symbol	Description	Exp. q-value: Combined	Sum PEP Score
P60709	ACTB	Actin, cytoplasmic 1	0	118.576
Q9UGM3	DMBT1	Deleted in malignant brain tumors 1 protein	0	96.483
Q9Y6R7	FCGBP	IgGFc-binding protein	0	83.984
Q9HC84	MUC5B	Mucin-5B	0	82.593
P01833	PIGR	Polymeric immunoglobulin receptor	0	74.675
P02768	ALB	Serum albumin	0	68.407
P01834	IGKC	Immunoglobulin kappa constant	0	65.497
P01876	IGHA1	Immunoglobulin heavy constant alpha 1	0	57.069
A0A0C4DGN4	ZG16B	Zymogen granule protein 16 homolog B	0	54.927
Q8N4F0	BPIFB2	BPI fold-containing family B member 2	0	51.825
P23280	CA6	Carbonic anhydrase 6	0	50.741
P07737	PFN1	Profilin-1	0	46.007
Q8TDL5	BPIFB1	BPI fold-containing family B member 1	0	43.776
P01871	IGHM	Immunoglobulin heavy constant mu	0	43.278
P04745	AMY1A	Alpha-amylase 1	0	43.225
B4DNK4	PKM	Pyruvate kinase	0	42.287
P01037	CST1	Cystatin-SN	0	39.741
A0A0G2JMB2	IGHA2	Immunoglobulin heavy constant alpha 2	0	38.534
P04406	GAPDH	Glyceraldehyde-3-phosphate dehydrogenase	0	37.911
P01036	CST4	Cystatin-S	0	37.331
P01024	C3	Complement C3	0	36.744
P06733	ENO1	Alpha-enolase	0	36.169
P12273	PIP	Prolactin-inducible protein	0	31.918
Q96DR5	BPIFA2	BPI fold-containing family A member 2	0	31.242
A8K2U0	A2ML1	Alpha-2-macroglobulin-like protein 1	0	29.477
P13796	LCP1	Plastin-2	0	28.462
P0DOY2	IGLC2	Immunoglobulin lambda constant 2	0	27.701
P01763	IGHV3-48	Immunoglobulin heavy variable 3-48	0	27.021
Q02413	DSG1	Desmoglein-1	0	26.666
P01857	IGHG1	Immunoglobulin heavy constant gamma 1	0	25.958
Q6P5S2	LEG1	Protein LEG1 homolog	0	25.85
P28325	CST5	Cystatin-D	0	24.277
P35579	MYH9	Myosin-9	0	24.102
P10909	CLU	Clusterin	0	23.626
A0A0G2JIW1	HSPA1B	Heat shock 70 kDa protein 1B	0	23.22
A0A024R6I7	SERPINA1	Alpha-1-antitrypsin	0	22.104
P01602	IGKV1-5	Immunoglobulin kappa variable 1-5	0	21.979
P32926	DSG3	Desmoglein-3	0	20.989

P63104	YWHAZ	14-3-3 protein zeta/delta	0	20.938
O00560	SDCBP	Syntenin-1	0	20.814
P01591	JCHAIN	Immunoglobulin J chain	0	20.496
P15924	DSP	Desmoplakin	0	19.903
A0A286YFJ8	IGHG4	Immunoglobulin heavy constant gamma 4	0	19.842
P18206	VCL	Vinculin	0	19.746
P01593	IGKV1D-33	Immunoglobulin kappa variable 1D-33	0	19.657
Q01469	FABP5	Fatty acid-binding protein, epidermal	0	19.263
P68871	HBB	Hemoglobin subunit beta	0	19.007
P02647	APOA1	Apolipoprotein A-I	0	18.964
P27487	DPP4	Dipeptidyl peptidase 4	0	18.658
P29508	SERPINB3	Serpin B3	0	18.46
P14923	JUP	Junction plakoglobin	0	18.264
P01619	IGKV3-20	Immunoglobulin kappa variable 3-20	0	17.958
P02671	FGA	Fibrinogen alpha chain	0	17.828
H7BZ94	P4HB	Protein disulfide-isomerase	0	17.792
K7EMN2	PGD	6-phosphogluconate dehydrogenase, decarboxylating	0	17.634
P31025	LCN1	Lipocalin-1	0	17.591
A0A286YEY4	IGHG2	Immunoglobulin heavy constant gamma 2	0	17.372
A0A0A0MT01	GSN	Gelsolin	0	16.862
P02814	SMR3B	Submaxillary gland androgen-regulated protein 3B	0	16.127
A0A0C4DH42	IGHV3-66	Immunoglobulin heavy variable 3-66	0	15.937
O43490	PROM1	Prominin-1	0	15.847
A0A1B0GU92		Uncharacterized protein	0	15.458
A8MX94	GSTP1	Glutathione S-transferase P	0	15.447
A0A0C4DH73	IGKV1-12	Immunoglobulin kappa variable 1-12	0	15.262
P35030	PRSS3	Trypsin-3	0	15.195
Q8NHM4	PRSS3P2	Putative trypsin-6	0	15.15
E7EQ64	PRSS1	Trypsin-1	0	14.884
P47929	LGALS7	Galectin-7	0	14.791
P04080	CSTB	Cystatin-B	0	14.499
P69905	HBA1	Hemoglobin subunit alpha	0	14.419
P01034	CST3	Cystatin-C	0	14.216
E9PLF4	HSPA8	Heat shock cognate 71 kDa protein	0	14.076
Q71U36	TUBA1A	Tubulin alpha-1A	0	13.649
G5EA09	SDCBP	Syndecan binding protein (Syntenin)	0	13.577
A0A0B4J231	IGLL5	Immunoglobulin lambda-like polypeptide 5	0	13.201
E7EQB2	LTF	Lactotransferrin	0	12.997
A0A0A0MRZ8	IGKV3D-11	Immunoglobulin kappa variable 3D-11	0	12.871
F5H5D3	TUBA1C	Tubulin alpha chain	0	12.326
P02656	APOC3	Apolipoprotein C-III	0	12.067
Q6MZM9	PRR27	Proline-rich protein 27	0	12.053

P04792	HSPB1	Heat shock protein beta-1	0	11.882
Q13421	MSLN	Mesothelin	0	11.586
C9JEU5	FGG	Fibrinogen gamma chain	0	10.916
M0R1V7	UBA52	Ubiquitin-60S ribosomal protein L40	0	10.862
Q6UWP8	SBSN	Suprabasin	0	10.287
P02675	FGB	Fibrinogen beta chain	0	10.209
P01782	IGHV3-9	Immunoglobulin heavy variable 3-9	0	9.924
Q08188	TGM3	Protein-glutamine gamma-glutamyltransferase E	0	9.639
Q8WUM4	PDCD6IP	Programmed cell death 6-interacting protein	0	9.439
P07900	HSP90AA1	Heat shock protein HSP 90-alpha	0	9.28
P17213	BPI	Bactericidal permeability-increasing protein	0	9.261
O75828	CBR3	Carbonyl reductase [NADPH] 3	0	9.253
P36952	SERPINB5	Serpin B5	0	9.103
A0A0C4DH38	IGHV5-51	Immunoglobulin heavy variable 5-51	0	9.032
G8JLG2	CDSN	Corneodesmosin	0	9.018
P06312	IGKV4-1	Immunoglobulin kappa variable 4-1	0	9.014
F5H386	LPO	Lactoperoxidase	0	8.821
P81605	DCD	Dermcidin	0	8.819
P30740	SERPINB1	Leukocyte elastase inhibitor	0	8.794
B7ZKJ8	ITIH4	Inter alpha-trypsin inhibitor, heavy chain 4	0	8.77
Q06830	PRDX1	Peroxiredoxin-1	0	8.568
A0A0B4J1V1	IGHV3-21	Immunoglobulin heavy variable 3-21	0	8.482
P59665	DEFA1	Neutrophil defensin 1	0	8.445
O60664	PLIN3	Perilipin-3	0	8.433
B7Z6Z4	MYL6	Myosin light polypeptide 6	0	8.374
Q5T749	KPRP	Keratinocyte proline-rich protein	0	8.297
P22314	UBA1	Ubiquitin-like modifier-activating enzyme 1	0	8.209
P01762	IGHV3-11	Immunoglobulin heavy variable 3-11	0	8.167
P01624	IGKV3-15	Immunoglobulin kappa variable 3-15	0	7.789
Q5T3N1	ANXA1	Annexin	0	7.737
Q15847	ADIRF	Adipogenesis regulatory factor	0	7.724
A0A075B7B8	IGHV3OR16-12	Immunoglobulin heavy variable 3/OR16-12	0	7.611
P04075	ALDOA	Fructose-bisphosphate aldolase A	0	7.102
Q13885	TUBB2A	Tubulin beta-2A chain	0	7.08
P14174	MIF	Macrophage migration inhibitory factor	0	6.858
D6RFL4	CD14	Monocyte differentiation antigen CD14	0	6.841
A0A075B7D4	IGKV1OR2-108	Immunoglobulin kappa variable 1/OR2-108	0	6.77
A0A0C4DH31	IGHV1-18	Immunoglobulin heavy variable 1-18	0	6.715
J3QLK0	RAC3	Ras-related C3 botulinum toxin substrate 3	0	6.469
Q13835	PKP1	Plakophilin-1	0	6.339
P20061	TCN1	Transcobalamin-1	0	6.323
P80188	LCN2	Neutrophil gelatinase-associated lipocalin	0	6.244

A0A0C4DH72	IGKV1-6	Immunoglobulin kappa variable 1-6	0	6.196
A0A0B4J259	LYZ	Lysozyme	0	6.174
P30044	PRDX5	Peroxiredoxin-5, mitochondrial	0	6.16
P31944	CASP14	Caspase-14	0	6.097
O15511	ARPC5	Actin-related protein 2/3 complex subunit 5	0	5.973
P00338	LDHA	L-lactate dehydrogenase A chain	0	5.929
P06870	KLK1	Kallikrein-1	0	5.639
F5GXS2	ACTN4	Alpha-actinin-4	0	5.619
E9PK25	CFL1	Cofilin-1	0	5.567
Q16778	HIST2H2BE	Histone H2B type 2-E	0	5.555
P01700	IGLV1-47	Immunoglobulin lambda variable 1-47	0	5.534
P05109	S100A8	Protein S100-A8	0	5.503
B4DEH5	LTA4H	Leukotriene A-4 hydrolase	0	5.313
P40199	CEACAM6	Carcinoembryonic antigen-related cell adhesion molecule 6	0	5.297
H3BRJ0	CORO1A	Coronin	0	5.196
A0A0B4J1R6	TKT	Transketolase	0	5.161
A0A0C4DH69	IGKV1-9	Immunoglobulin kappa variable 1-9	0	5.09
Q9UBG3	CRNN	Cornulin	0	4.974
P19971	TYMP	Thymidine phosphorylase	0	4.959
X6RJP6	TAGLN2	Transgelin-2	0	4.848
P58546	MTPN	Myotrophin	0	4.716
P10599	TXN	Thioredoxin	0	4.665
K7ESA3	VAT1	Synaptic vesicle membrane protein VAT-1 homolog	0	4.588
Q9HCY8	S100A14	Protein S100-A14	0	4.483
Q9GZZ8	LACRT	Extracellular glycoprotein lacritin	0	4.42
P55058	PLTP	Phospholipid transfer protein	0	4.416
P35321	SPRR1A	Cornifin-A	0	4.363
P22532	SPRR2D	Small proline-rich protein 2D	0	4.255
A0A087WZM5	FKBP1A	Peptidylprolyl isomerase	0	4.252
F5H6T1	ACTR2	Actin-related protein 2 homolog	0	4.234
P06702	S100A9	Protein S100-A9	0	4.212
A0A087WW87	IGKV2-40	Immunoglobulin kappa variable 2-40	0	4.199
P11021	HSPA5	Endoplasmic reticulum chaperone BiP	0	4.194
P80748	IGLV3-21	Immunoglobulin lambda variable 3-21	0	4.19
P48595	SERPINB10	Serpin B10	0	4.177
A0A075B6S9	IGKV1-37	Immunoglobulin kappa variable 1-37	0	4.174
P01714	IGLV3-19	Immunoglobulin lambda variable 3-19	0	4.164
E9PD92	G6PD	Glucose-6-phosphate 1-dehydrogenase	0	3.999
P62805	HISTH4A	Histone H4	0	3.984
E9PG15	YWHAQ	14-3-3 protein theta	0	3.974
P02042	HBD	Hemoglobin subunit delta	0	3.972
C9JF17	APOD	Apolipoprotein D	0	3.931

F5GXS0	C4B	Complement C4-B	0	3.883
K7EIT4	YWHAE	14-3-3 protein epsilon	0	3.878
Q16777	HIST2H2AC	Histone H2A type 2-C	0	3.837
Q01518	CAP1	Adenylyl cyclase-associated protein 1	0	3.825
Q9Y490	TLN1	Talin-1	0	3.79
P02790	HPX	Hemopexin	0	3.773
P31947	SFN	14-3-3 protein sigma	0	3.771
P01023	A2M	Alpha-2-macroglobulin	0	3.725
B1AKY9	ATP1A2	Sodium/potassium-transporting ATPase subunit alpha	0	3.713
P06703	S100A6	Protein S100-A6	0	3.593
A0A1W2PRQ0	FAM49B	CYFIP-related Rac1 interactor B	0	3.518
E9PKU7	GANAB	Neutral alpha-glucosidase AB	0	3.508
F8VWK8	CD63	Tetraspanin	0	3.505
E9PNQ8	THY1	Thy-1 membrane glycoprotein	0	3.503
O75083	WDR1	WD repeat-containing protein 1	0	3.501
A0A0B4J2D9	IGKV1D-13	Immunoglobulin kappa variable 1D-13	0	3.481
P08311	CTSG	Cathepsin G	0	3.48
P40121	CAPG	Macrophage-capping protein	0	3.46
Q5H9B4	TIMP1	Metalloproteinase inhibitor 1	0	3.454
P29373	CRABP2	Cellular retinoic acid-binding protein 2	0	3.447
P52790	HK3	Hexokinase-3	0	3.431
E9PLE9	NUCB2	Nucleobindin-2	0	3.393
E9PLZ3	RNH1	Ribonuclease inhibitor	0	3.348
Q5D862	FLG2	Filaggrin-2	0	3.273
B3EWG6	FAM25G	Protein FAM25G	0	3.243
P60174-1	TPI1	Triosephosphate isomerase	0	3.238
Q9H3Z4	DNAJC5	DnaJ homolog subfamily C member 5	0	3.197
B5MDF5	RAN	GTP-binding nuclear protein Ran	0	3.189
P40926	MDH2	Malate dehydrogenase, mitochondrial	0	3.153
C9J0E4	CSTA	Cystatin-A	0	3.125
P61266	STX1B	Syntaxin-1B	0	3.122
P21333	FLNA	Filamin-A	0	3.099
A0A087WT87	SLC1A3	Amino acid transporter	0	3.09
P03973	SLPI	Antileukoproteinase	0	3.054
P33908	MAN1A1	Mannosyl-oligosaccharide 1,2-alpha-mannosidase IA	0	3.045
H0YGX7	ARHGDIB	Rho GDP-dissociation inhibitor 2	0	3.026
P50395	GDI2	Rab GDP dissociation inhibitor beta	0	3.017
M0R261	PGLS	6-phosphogluconolactonase	0	3.012
C9JL75	TMEM94	Transmembrane protein 94	0	2.969
P30043	BLVRB	Flavin reductase (NADPH)	0	2.963
P00558	PGK1	Phosphoglycerate kinase 1	0	2.913
P22735	TGM1	Protein-glutamine gamma-glutamyltransferase K	0	2.771

E9PI80	CD59	CD59 glycoprotein	0	2.765
P55064	AQP5	Aquaporin-5	0	2.75
Q6ZVX7	NCCRP1	F-box only protein 50	0	2.628
Q08380	LGALS3BP	Galectin-3-binding protein	0	2.623
E9PP11	NPEPPS	Puromycin-sensitive aminopeptidase	0	2.614
A0A0A0MT26	ATP1A3	Sodium/potassium-transporting ATPase subunit alpha-3	0	2.598
A0A024R0K5	CEACAM5	Carcinoembryonic antigen-related cell adhesion molecule 5	0	2.593
E9PFP8	PCBP3	Poly(rC)-binding protein 3	0	2.52
P04040	CAT	Catalase	0	2.401
P22894	MMP8	Neutrophil collagenase	0	2.397
P60880	SNAP25	Synaptosomal-associated protein 25	0	2.358
P14780	MMP9	Matrix metalloproteinase-9	0	2.343
Q9HAV4	XPO5	Exportin-5	0	2.333
Q96P63	SERPINB12	Serpin B12	0.002	2.288
P00491	PNP	Purine nucleoside phosphorylase	0.002	2.288
P16870	CPE	Carboxypeptidase E	0.002	2.274
P59998	ARPC4	Actin-related protein 2/3 complex subunit 4	0.002	2.226
Q05639	EEF1A2	Elongation factor 1-alpha 2	0.002	2.21
A0A0D9SF54	SPTAN1	Spectrin alpha chain, non-erythrocytic 1	0.002	2.191
P22528	SPRR1B	Cornifin-B	0.002	2.184
P30041	PRDX6	Peroxiredoxin-6	0.002	2.155
A8MXZ4	GPRC5	G-protein-coupled receptor family C group 5 member C	0.002	2.127
B1AN48	SPRR3	Small proline-rich protein 3	0.002	2.126
H0Y7L5	APOE	Apolipoprotein E	0.002	2.117
P61158	ACTR3	Actin-related protein	0.002	2.103
P08590	MYL3	Myosin light chain 3	0.002	2.069
E9PFZ2	CP	Ceruloplasmin	0.002	2.056
P01742	IGHV1-69	Immunoglobulin heavy variable 1-69	0.002	2.056
O00391	QSOX1	Sulfhydryl oxidase 1	0.002	2.052
A8MVZ9	ALDOC	Fructose-bisphosphate aldolase	0.002	2.017
P60201	PLP1	Myelin proteolipid protein	0.002	2.016
E9PC69	MARK2	Non-specific serine/threonine protein kinase	0.002	2.007
P62873	GNB1	Guanine nucleotide-binding protein G(I)/G(S)/G(T) subunit beta-1	0.018	1.975
X6R3S7	TFF3	Trefoil factor 3	0.018	1.95
P00738	HP	Haptoglobin	0.018	1.92
M0R0R2	RPS5	40S ribosomal protein S5	0.018	1.856
P08865	RPSA	40S ribosomal protein SA	0.018	1.85
Q9H0L4	CSTF2T	Cleavage stimulation factor subunit 2 tau variant	0.017	1.814
Q16799	RTN1	Reticulon-1	0.017	1.809
Q5BJH1	PSAP	Prosaposin	0.017	1.806
A0A075B6I0	IGLV8-61	Immunoglobulin lambda variable 8-61	0.017	1.787

P62491	RAB11A	Ras-related protein Rab-11A	0.017	1.774
P27482	CALML3	Calmodulin-like protein 3	0.017	1.771
P04899	GNAI2	Guanine nucleotide-binding protein G(i) subunit alpha-2	0.017	1.763
C9J4N6	IDH1	Isocitrate dehydrogenase [NADP] cytoplasmic	0.017	1.737
P78417	GSTO1	Glutathione S-transferase omega-1	0.017	1.727
P12107	COL11A1	Collagen alpha-1(XI) chain	0.017	1.724
A8MUD9	RPL7	60S ribosomal protein L7	0.017	1.706
P15531	NME1	Nucleoside diphosphate kinase A	0.017	1.703
O14745	SLC9A3R1	Na(+)/H(+) exchange regulatory cofactor NHE-RF1	0.018	1.697
A0A075B6I9	IGLV7-46	Immunoglobulin lambda variable 7-46	0.017	1.658
Q13637	RAB32	Ras-related protein Rab-32	0.017	1.63
E9PDP5	ANKHD1	Ankyrin repeat and KH domain-containing protein 1	0.017	1.618
P52907	CAPZA1	F-actin-capping protein subunit alpha-1	0.017	1.607
Q96L46	CAPNS2	Calpain small subunit 2	0.017	1.6
C9JV77	AHSG	Alpha-2-HS-glycoprotein	0.017	1.596
Q14CN2	CLCA4	Calcium-activated chloride channel regulator 4	0.017	1.59
Q14974	KPNB1	Importin subunit beta-1	0.019	1.576
H0YHA7	RPL18	60S ribosomal protein L18	0.019	1.563
Q13191	CBLB	E3 ubiquitin-protein ligase CBL-B	0.019	1.532
P06727	APOA4	Apolipoprotein A-IV	0.019	1.53
E7EN89	TOLLIP	Toll interacting protein	0.02	1.49
O94989	ARHGEF15	Rho guanine nucleotide exchange factor 15	0.021	1.447
P19823	ITIH2	Inter-alpha-trypsin inhibitor heavy chain H2	0.021	1.438
P23490	LOR	Loricrin	0.021	1.425
A0A087WUI2	HNRNPA2	Heterogeneous nuclear ribonucleoproteins A2/B1	0.034	1.371
C9JEE0	IGLL1	Immunoglobulin lambda-like polypeptide 1	0.034	1.366
P39880	CUX1	Homeobox protein cut-like 1	0.034	1.364
A0A0B4J1V6	IGHV3-73	Immunoglobulin heavy variable 3-73	0.036	1.33
H0YNP5	ANXA2	Annexin	0.036	1.327
Q93073	SECISBP2L	Selenocysteine insertion sequence-binding protein 2-like	0.036	1.32
A0A0C4DGY1	TMPRSS9	Transmembrane protease serine 9	0.036	1.305
Q7Z7M9	GALNT5	Polypeptide N-acetylgalactosaminyltransferase 5	0.035	1.291
Q14966	ZNF638	Zinc finger protein 638	0.035	1.268
P11216	PYGB	Glycogen phosphorylase, brain form	0.038	1.243
M0QXA7	WIZ	Protein Wiz	0.046	1.224

Supplementary Table S8. *Significantly changed Discovery group miRNAs.*

miRNA	Fold Change	p-value
664b-5p	2.10	0.098
642a-5p	1.80	0.001
3140-5p	1.75	0.046
380-3p	1.64	0.048
513c-5p	1.58	0.026
1248	1.56	0.063
134-3p	1.50	0.058
1-5p	1.49	0.066
3131	1.47	0.017
1257	1.45	0.002
376b-3p	1.43	0.014
126-3p	1.40	0.071
3p	1.37	0.086
128-2-5p	1.33	0.040
96-5p	1.28	0.066
1269a	1.27	0.096
18b-5p	-1.25	0.019
3185	-1.26	0.016
329-3p	-1.28	0.008
1255a	-1.28	0.009
518e-3p	-1.28	0.003
219a-2-3p	-1.35	0.007

Supplementary Table S9. *Discovery group miRNAs that change with PoMS TMD.*

miRNA	Fold change	p-value
664b-5p	2.1	0.0985
642a-5p	1.9	0.0008
1304-3p	1.88	0.02
3140-5p	1.75	0.0465
380-3p	1.64	0.0484
513c-5p	1.58	0.026
1248	1.56	0.0633
134-3p	1.5	0.058
1-5p	1.49	0.0661
3131	1.47	0.0167
1257	1.45	0.0019
519d-3p	1.43	0.04
376b-3p	1.43	0.0144
126-3p	1.4	0.0715
128-2-5p	1.33	0.0404
381-3p	1.3	0.01
374b-5p	1.29	0.05
96-5p	1.28	0.0662
1269a	1.27	0.0956
532-3p	1.27	0.02
873-3p	1.24	0.03
1296-3p	1.22	0.03
24-3p	1.2	0.04
29a-3p	1.08	0.01
181b-5p + 181d-5p*	-1.25	0.0192
3185	-1.27	0.0157
329-3p	-1.28	0.0076
1255a	-1.28	0.0086
765	-1.28	0.05
219a-2-3p	-1.35	0.0066
221-3p	-1.52	0.01

*Two miRNA that are indistinguishable from each other

Supplementary Table S10. *Discovery group miRNAs that change with PoMS FI subscale.*

miRNA	Fold change	p-value
619-3p	2.15	0.0063
140-5p	2.01	0.0006
98-5p	1.87	0.0138
4536-5p	1.85	0.0059
105-5p	1.79	0.0127
485-5p	1.74	0.0142
1245a	1.6	0.009
410-3p	1.59	0.0403
654-3p	1.57	0.0108
142-3p	1.55	0.0357
214-3p	1.53	0.0102
200c-3p	1.51	0.0369
548e-3p	1.5	0.034
3147	1.47	0.0367
30e-5p	1.46	0.0057
509-3-5p	1.36	0.0089
339-5p	1.34	0.0248
1270	1.34	0.0285
503-3p	1.32	0.0176
1289	1.32	0.0407
660-3p	1.31	0.014
203a-3p	1.3	0.0389
18b-5p	1.29	0.0064
497-5p	0.77	0.0404
210-3p	-1.21	0.0273
181a-2-3p	-1.31	0.0278
1910-3p	-1.32	0.0407
598-3p	-1.33	0.0298
371a-5p	-1.43	0.0338
2110	-1.44	0.0293
655-3p	-1.46	0.0328
511-5p	-1.48	0.0337
663a	-1.5	0.0348
4454 + 7975*	-1.64	0.0102
614	-1.71	0.0138
182-5p	-1.77	0.0285

*Two miRNA that are indistinguishable from each other
